# Efficacy of pars plana vitrectomy combined with internal limiting membrane peeling and gas tamponade for treating myopic foveoschisis: a meta-analysis

**DOI:** 10.1186/s12886-024-03534-2

**Published:** 2024-07-18

**Authors:** Shuqi Song, Guanglun He, Di Huang, Xiaojuan Li, Zhenzhen Wu, Yongfeng Sun

**Affiliations:** Department of Ophthalmology, The Hospital of Beijing Municipal Corps, Chinese people’s Armed police Force, Northwest corner of Changhong Bridge Sanlitun, East Third Ring Road, Chaoyang District, Beijing, 100027 China

**Keywords:** Macular foveoschisis, Pars plana vitrectomy, Internal limiting membrane peeling, Gas tamponade

## Abstract

**Objective:**

This study aimed to evaluate and explore the efficacy of pars plana vitrectomy (PPV) combined with internal limiting membrane (ILM) peeling and gas tamponade in treating myopic foveoschisis (MF) through a meta-analysis.

**Methods:**

Systematic searches were conducted on the PubMed, Web of Science and National Library of Medicine (NLM) English-language databases and the China National Knowledge Infrastructure (CNKI) and Wanfang Chinese-language databases. The primary outcome measures were postoperative best-corrected visual acuity (BCVA) and central foveal thickness (CFT), with the secondary outcome being the postoperative complication rate. Data analysis was performed using RevMan5.3 software.

**Results:**

A total of 10 studies involving 234 eyes were included. The meta-analysis results showed the following: (1) The average postoperative BCVA improved compared with preoperative levels, with an average improvement in the logarithm of the minimum angle of resolution of 0.40, a statistically significant difference (95% CI: −0.44, − 0.20, *p <* 0.001); (2) the rate of postoperative BCVA improvement was 77% (95% CI: 65%, 90%, *p <* 0.001); (3) the postoperative CFT significantly decreased by an average of 385.92 μm, a statistically significant difference (95% CI: −437.85, − 333.98, *p <* 0.001); (4) the postoperative macular retinal complete reattachment rate was 90% (95% CI: 83%, 97%, *p <* 0.001); (5) the most common postoperative complication was a cataract, with an incidence of 55.9%.

**Conclusion:**

Using PPV combined with ILM peeling and gas tamponade to treat MF is reliable.

## Introduction

Myopic foveoschisis (MF), also known as myopic traction maculopathy, is a schisis-like thickening of the retina in eyes with high myopia with posterior staphyloma. The pathologic features may also include lamellar or full-thickness macular holes (FTMH), shallow foveal detachments (FD) and inner retinal fluid [1]. Myopic foveoschisis is a common pathological change in patients with high myopia, with a prevalence of 8–34% in this population. The condition can lead to significant visual impairment and distortion of vision and even affect the quality of life of the patient [1]. The onset of MF is often insidious and the condition progresses slowly. During its natural course, some patients may remain stable for a time, but most patients with MF may subsequently develop FD or FTMH, or both complications concurrently [2, 3].

Since 1957, macular scleral buckling surgery has been clinically applied for the treatment of MF. Research indicates that this surgical approach can alleviate the tension caused by eyeball expansion, improve abnormal eyeball structures and achieve a good postoperative retinal reattachment rate. However, it also poses risks, such as damage to peripheral eyeball structures, excessive compression range, scleral perforation and high incidence of subretinal haemorrhage complications, with the procedure being challenging to perform [4]. In 2002, pars plana vitrectomy (PPV) was introduced for treating MF, aiming to relieve the vitreoretinal traction by removing the vitreous [5]. The prevailing approach at the time was to adopt a conservative observation first, operating only when complications arose and preserving the integrity of the internal limiting membrane (ILM) to avoid iatrogenic retinal hole formation. However, the surgical outcome under this perspective was highly unpredictable and prone to recurrence [6]. In recent years, some scholars have recommended proactive early intervention, which involves surgery before complications such as macular holes (MH) arise and adopting a treatment plan combining PPV with ILM peeling [7]. Although there are studies discussing the efficacy of this procedure, the standard surgical protocol for MF remains controversial, with many scholars concerned about intraoperative or postoperative iatrogenic hole formation.

Thus, this study employs a meta-analysis to systematically evaluate the efficacy of PPV combined with ILM peeling and gas tamponade in treating MF, aiming to provide evidence-based guidance for early intervention in MF treatment.

## Materials and methods

### Literature retrieval strategy

Following the Preferred Reporting Items for Systematic Reviews and Meta-Analyses guidelines, systematic searches were conducted on the PubMed, Web of Science, NLM, CNKI and Wanfang databases. The search timeline was from the inception of these databases to 1 January 2023. A combination of subject terms and free words were used for the retrieval: ‘internal limiting membrana OR ILM’ AND ‘vitrectomy OR PPV OR pars plana vitrectomy’ AND ‘myopic foveoschisis OR foveoschisis OR retinoschisis OR maculophathy’. The studies included were all published in English and whose journals were ranked in the top 25% of SCI studies.

### Inclusion and exclusion criteria

The inclusion criteria were as follows: (1) Study type: retrospective or prospective studies; (2) study subjects: highly myopic patients (refractive error > − 6.0 D, axial length > 26 mm) with a confirmed diagnosis of foveoschisis via optical coherence tomography (OCT) presenting with complaints of decreased vision or distorted vision; (3) intervention: surgical intervention involving PPV combined with ILM peeling and gas tamponade; (4) outcome measures: best-corrected visual acuity (BCVA), central foveal thickness (CFT) and postoperative complication rate.

The exclusion criteria were as follows: (1) Patients with other ocular diseases affecting vision and visual field, such as MH, retinal detachment (RD), choroidal neovascularisation (CNV), macular atrophy, ocular trauma or those who underwent PPV or retinal laser therapy; (2) use of fillers other than gas; (3) studies with < 20 participants or no specific outcome measurement data; (4) studies with a follow-up period of < 6 months; (5) duplicated studies.

### Literature screening and data extraction

Two researchers independently screened the literature. Preliminary screening was performed based on titles and abstracts, followed by a full-text review according to the inclusion and exclusion criteria. In cases of disagreement, the opinion of a third researcher was sought, and a consensus was reached through discussion. After the literature screening, two researchers independently extracted data, which included the following details: first author, year of publication, country of publication, study type, number of affected eyes, surgical fill materials used, follow-up duration, refractive error and axial length.

### Intervention procedures

All patients underwent a thorough vitrectomy, followed by the staining and peeling of the ILM in the macular region. At the end of the surgery, either air or an inert gas was infused into the eye. For patients with significant cataracts, concurrent cataract phacoemulsification and intraocular lens implantation were performed during the procedure.

### Outcome measures

Primary outcome measures included the following: (1) The degree of retinal reattachment represented by CFT measurement, with complete structural recovery indicating that the retinal layer in the macular area was entirely reattached, and no hypo-reflective zones were observed in OCT scans; (2) BCVA, represented by the logarithm of the minimum angle of resolution (logMAR). The secondary outcome measure was the incidence of postoperative complications. All outcome measures used data from the last follow-up visit.

### Quality assessment of literature

The Newcastle–Ottawa Scale was employed to evaluate the quality of the finally included cohort studies and case-control studies, assessing in terms of selection, comparability and either exposure or outcome. The total score for this scale is 9 points, with studies scoring < 5 being considered low quality and those scoring ≥ 5 being considered high quality [8].

### Statistical analysis method

Meta-analysis was conducted using STATA (version 17.0) software. Continuous variables were represented using the standardised mean difference (SMD) while categorical variables employed the odds ratio as the effect estimate. Both types of estimates were presented as point estimates with 95% CIs. Heterogeneity among the studies was evaluated using the *I*^*2*^ test. If *I*^*2*^ < 50% or *p >* 0.1, studies were considered homogenous and a fixed-effects model (Mantel–Haenszel) was applied. If *I*^*2*^ > 50% or *p* ≤ 0.1, indicating significant heterogeneity among studies, a random-effects model (DerSimonian–Laird) was used. In this study, subgroup analysis was used to explore possible sources of heterogeneity, and the Q test was used for subgroup analysis. Egger’s test is used to check publication bias.The significance level for the meta-analysis was set at *α* = 0.05.

## Results

### Literature retrieval results

**Fig. 1:**
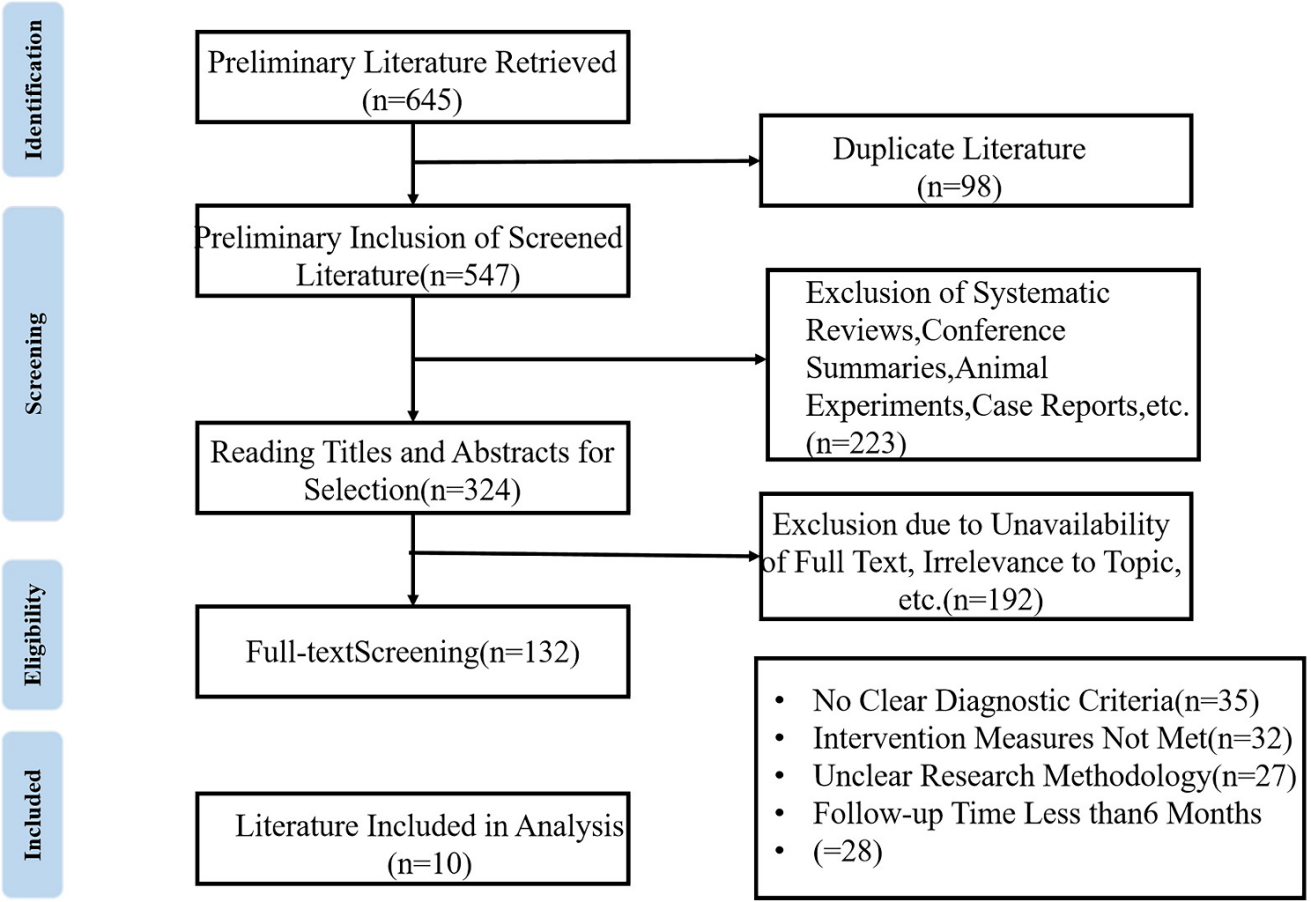
Literature screening flow chart

A total of 645 articles were identified through database searching. After excluding 98 duplicates and 223 studies such as systematic reviews and case reports, 10 articles [9–18] were ultimately included for meta-analysis based on the inclusion and exclusion criteria and after full-text assessment for studies with unclear diagnostic criteria and non-conforming interventions. The literature selection flowchart is shown in Fig. 1.

### Basic characteristics and quality assessment of included literature

Of the 10 final articles, which were published between 2012 and 2022, 6 were retrospective cohort studies and 4 were prospective cohort studies. A total of 234 eyes were included. The surgical methods were essentially consistent. After vitrectomy, the ILM was stained with either brilliant blue G (BBG), indocyanine green (ICG) or triamcinolone acetonide (TA), with the choice of dye left to the surgeon’s discretion [19]. The ILM peeling range was between the superior and inferior vascular arcades. After the surgery, the vitreous cavity was filled with perfluoropropane (C_3_F_8_), sulfur hexafluoride (SF_6_), hexafluoroethane (C_2_F_6_) or air, with the choice of filler also left to the surgeon’s discretion. All patients were advised to maintain a prone position for at least 1 week postoperatively.

The quality assessment scores of the included studies ranged between 6 and 8, indicating medium- to high-quality research. The basic characteristics and quality assessment results of the included literature are detailed in Table 1.

**Table 1:** Basic characteristics of included research and evaluation level of literature quality

Literature included	Year of publication	Country of publication	Research type	Number of diseased eyes	Filling materials	Staining Method	diopter	Eye axis	Follow up time	Scoring of literature quality
Rizzo [9]	2019	Italy	Retrospective cohort study	25	air	BBG	-9.8 ± 0.5	29.53 ± 1.55	12	7
Elwan [10]	2019	Egypt	Prospective cohort study	15	air	BBG	>-8.0	29.15 ± 11.87	18	8
Al-Badawi [11]	2019	Egypt	Prospective cohort study	11	air	TA	-13.4 ± 2.7	29.7 ± 0.9	6	6
Iida [12]	2013	Japan	Retrospective cohort study	11	SF_6_	ICG	-13.2 ± 3.56	28.65 ± 1.76	15.9 ± 4.5	7
Figueroa [13]	2015	Spain	Retrospective cohort study	30	C_3_F_8_	BBG	-14.8 ± 4.4	-	33.8 ± 13	8
Kim [14]	2012	Korea	Prospective cohort study	9	C_3_F_8_	ICG	-18.21 ± 4.36	29.31 ± 1.10	12	8
Yun [15]	2017	China	Retrospective cohort study	40	air	Not clear	-13.1~-3.7	28.87 ± 0.33	12	8
Fujimoto [16]	2013	Japan	Prospective cohort study	17	C_3_F_8_/ SF_6_	ICG	-11.9 ± 4.1	29.7 ± 1.5	12	7
Zhang(1) [17]	2022	China	Prospective cohort study	28	C_3_F_8_	ICG	-13.09 ± 5.37	29.31 ± 0.99	16.74 ± 9.40	8
Zhang(2) [17]	2022	China	Prospective cohort study	11	C_2_F_6_	ICG				
Zhang(3) [17]	2022	China	Prospective cohort study	16	air	ICG				
Gui [18]	2020	China	Prospective cohort study	21	air	ICG	-11.1 ± 4.9	28.9 ± 2.1	18.7 ± 13.9	7

*Note* BBG = brilliant blue G; IGG = indocyanine green; TA = triamcinolone acetonide

### Meta-analysis results

#### Visual acuity improvement

##### (1) Best-corrected visual acuity difference

**Fig. 2:**
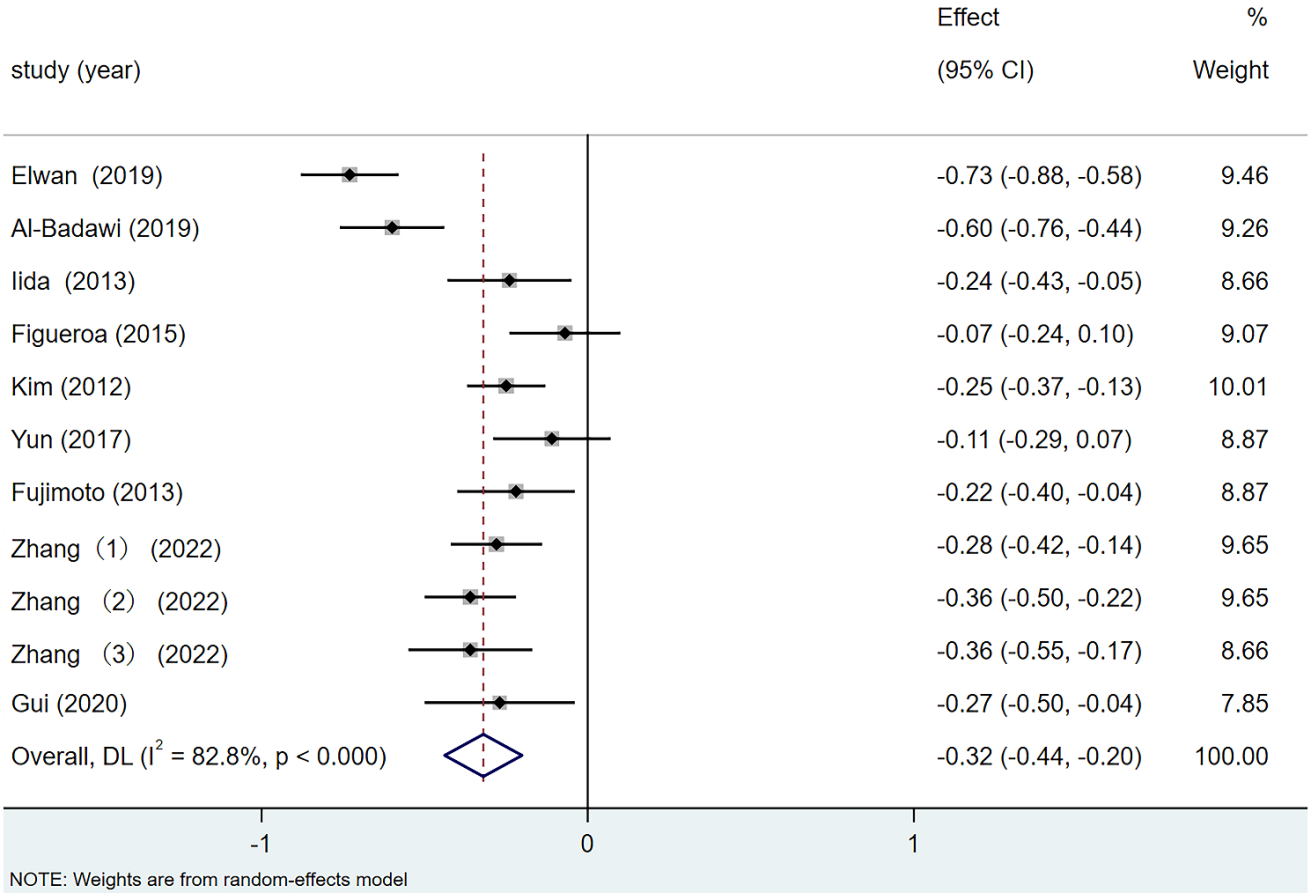
Forest plot of BCVA difference

Nine of the included articles reported preoperative and postoperative BCVA data. The meta-analysis revealed heterogeneity among the studies (I^2^ = 82.8%). Using a random-effects model for the meta-analysis, as shown in Fig. 2, the results indicated a statistically significant improvement in average postoperative BCVA compared with preoperative values (SMD = − 0.32, 95% CI: −0.44, − 0.20, *p <* 0.001).

##### (2) Postoperative best-corrected visual acuity difference improvement rate

**Fig. 3:**
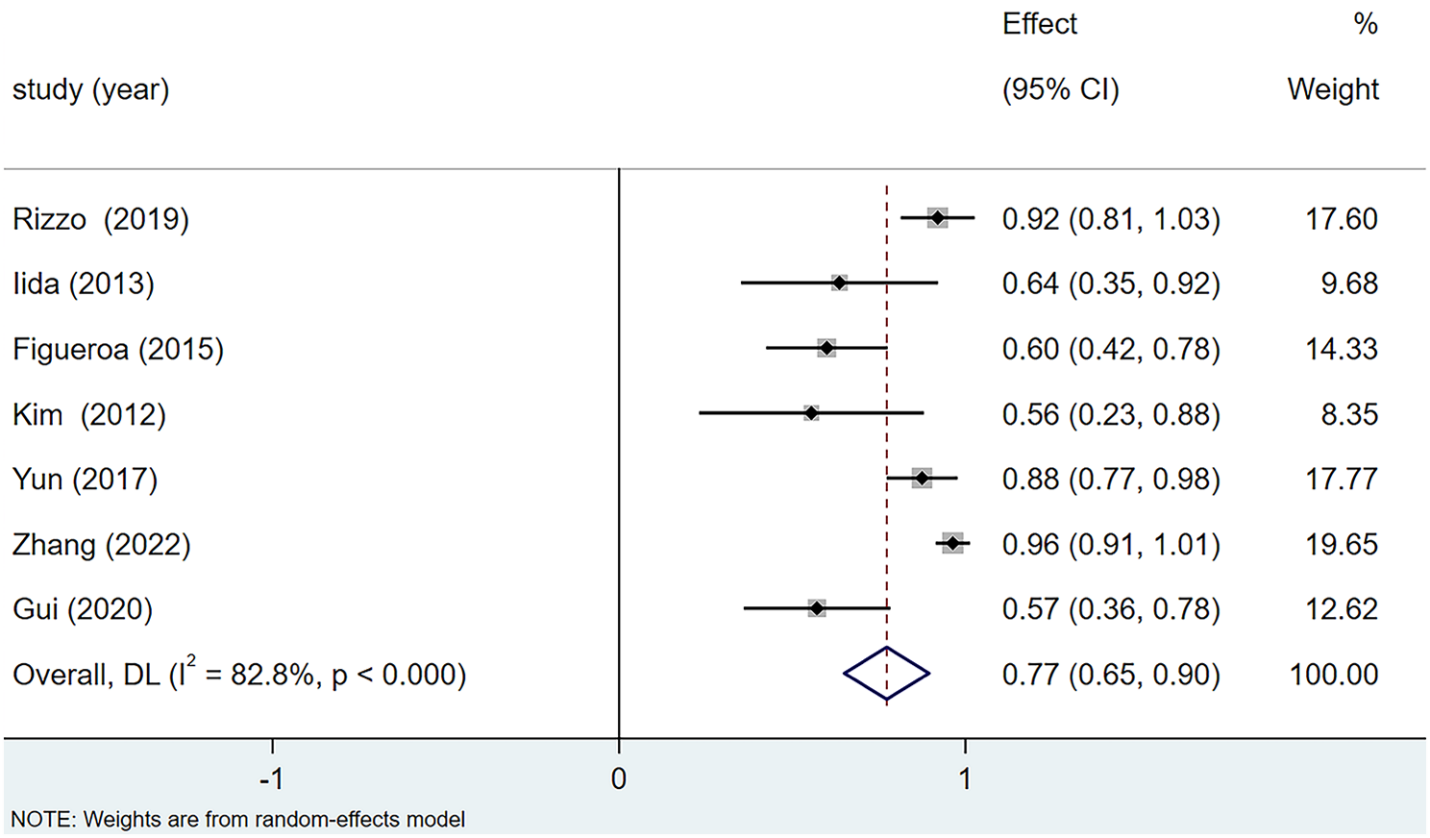
Forest plot of BCVA improvement rate

Out of eight articles that reported the postoperative BCVA improvement rate, the study by Elwan et al. [10] had an effect size of 1 and was thus not included in the meta-analysis. There was heterogeneity among the studies (I^2^ = 82.8%). Using a random-effects model for the meta-analysis, as shown in Fig. 3, the results indicated that the postoperative BCVA improvement rate after PPV combined with ILM peeling and gas tamponade was 77% (95% CI: 65%, 90%, *p <* 0.001).

#### Retinal flattening

##### (1) Central foveal thickness difference

**Fig. 4:**
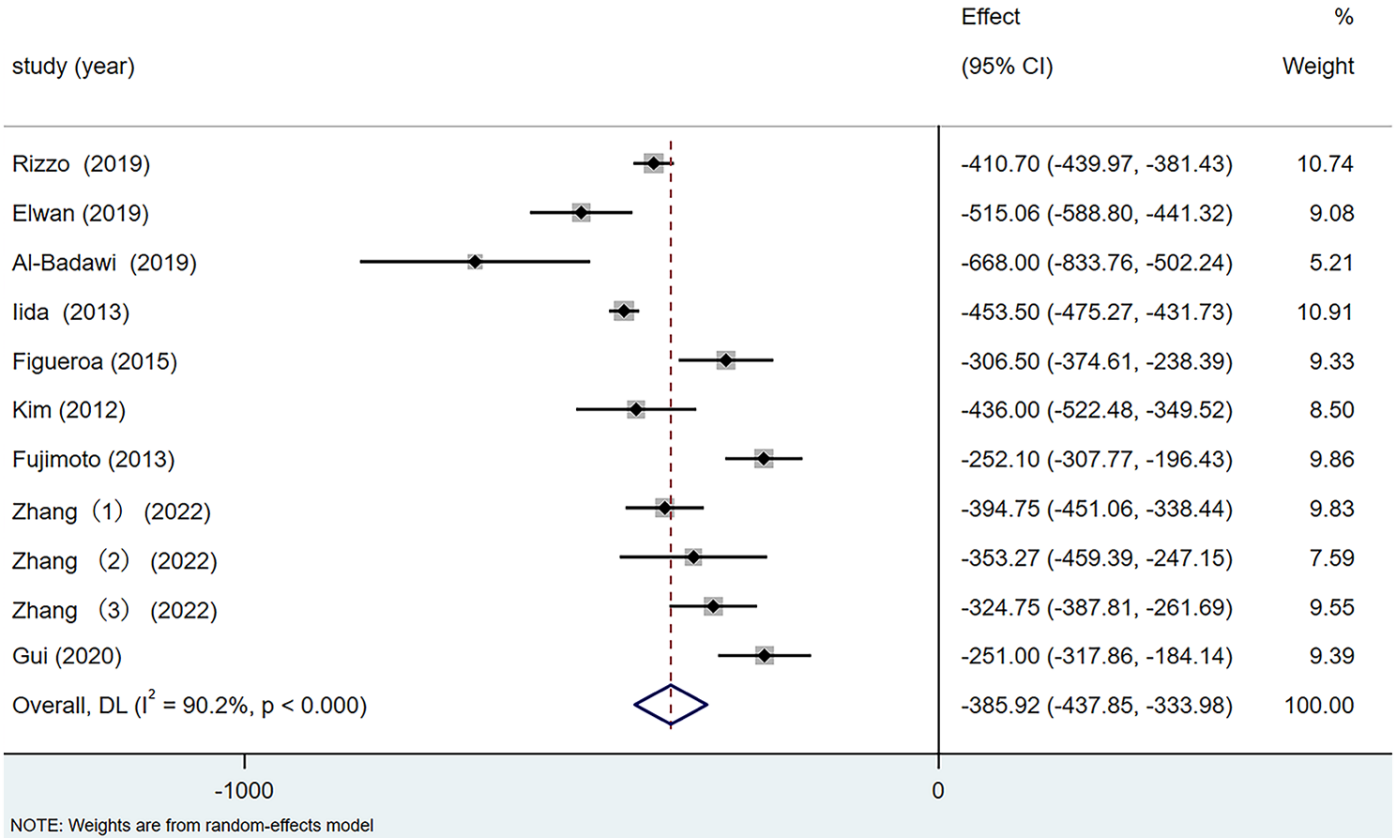
Forest plot of CFT difference

Nine articles reported preoperative and postoperative CFT data, with all included cases showing a decrease in macular retinal thickness. The meta-analysis revealed heterogeneity among the studies (I^2^ = 90.2%). Using a random-effects model, as shown in Fig. 4, the results showed a significant average reduction of 385.92 μm in postoperative CFT compared with preoperative measurements (95% CI: −437.85, − 333.98, *p <* 0.001).

##### (2) Postoperative macular retinal complete reattachment rate

**Fig. 5:**
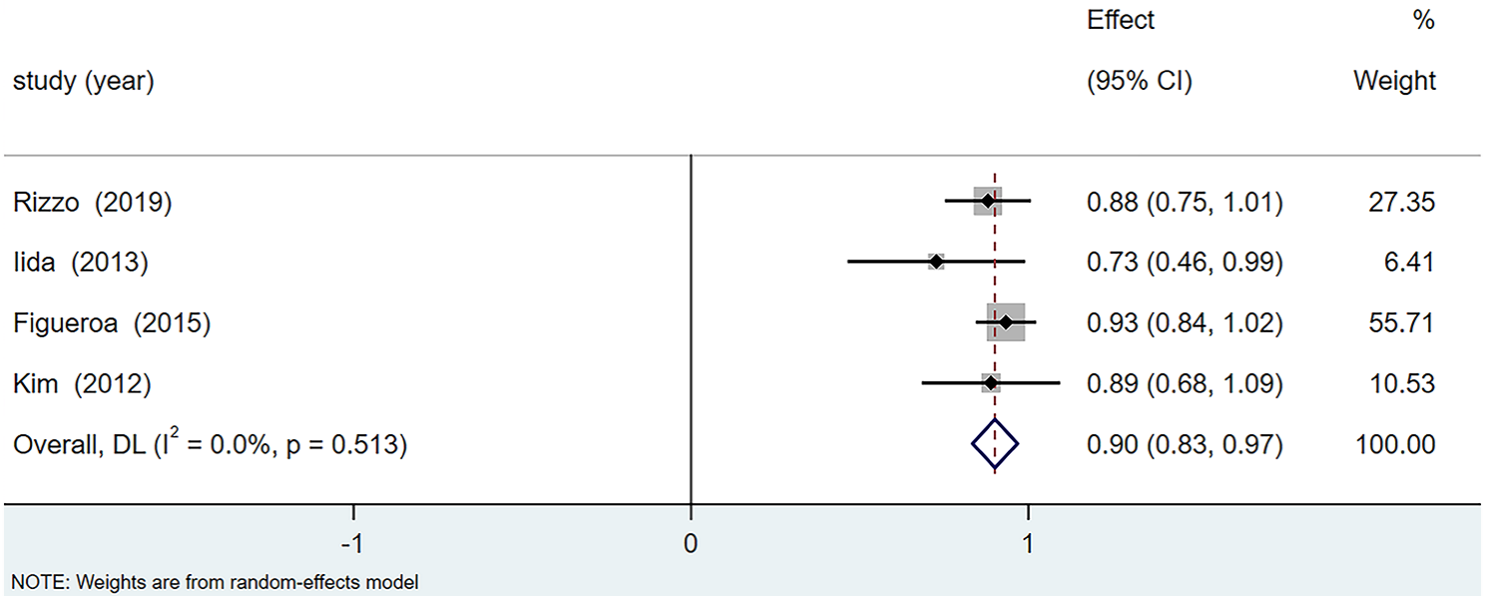
Forest plot of CFT improvement rate

Out of six articles reporting the postoperative CFT improvement rate, the studies by both Al-Badawi [11] and Yun et al. [15] had an effect size of 1 and thus were not included in the meta-analysis. The studies were homogeneous (I^2^ = 0.0%). Using a fixed-effects model, as shown in Fig. 5, the results indicated that the complete macular retinal reattachment after PPV combined with ILM peeling and gas tamponade was 90% (95% CI: 83%, 97%, *p <* 0.001).

### Postoperative complication incidence

The postoperative complication outcomes are detailed in Table 2. Neither Al-Badaw et al. [11] nor Fujimoto et al. [16] mentioned postoperative complications. Among the other articles, the most common postoperative complication was cataracts, with an incidence of 55.9%, followed by FTMH at 2.82% and RD at 2.35%. Other less common complications included CNV, chorioretinal atrophy and transient elevated intraocular pressure. Patients who developed FTMH, RD or macular hole RD (MHRD) postoperatively achieved retinal reattachment after a second PPV, but the visual prognosis was not favourable. Transient elevated intraocular pressure observed postoperatively could be effectively managed with medication [11].

**Table 2:** The occurrence of postoperative complications

Literature included	Number of diseased eyes	FTMH	RD	MHRD	Cataract	other
Rizzo	25	2	-	-	-	-
Elwan	15	-	-	-	3(9)	2
Iida	11	-	1	2		-
Figueroa	30	1	2		12(18)	-
Kim	9	1	-	1	-	-
Yun	40	-	-	-	-	-
Zhang	62	2	-		18(32)	-
Gui	21	-	2	-		-
Total	213	6	5	3	33(59)	2
Incidence	-	2.82%	2.35%	1.41%	55.93%	0.94%

*Note* ① - represents no occurrence; ②FTMH = Full-Thickness Macular Hole; RD = Retinal detachment; MHRD = Macular Hole Retinal Detachment

### Subgroup analysis

Since many factors during surgery, such as vitreous cavity fillings, filling materials and ILM staining materials, may affect the outcome, subgroup analysis was conducted in this study to explore the sources of heterogeneity. The heterogeneity sources in BCVA difference, postoperative BCVA improvement rate and CFT difference were explored using filling materials and ILM dyeing materials as grouping variables.

### Best-corrected visual acuity difference

**Fig. 6:**
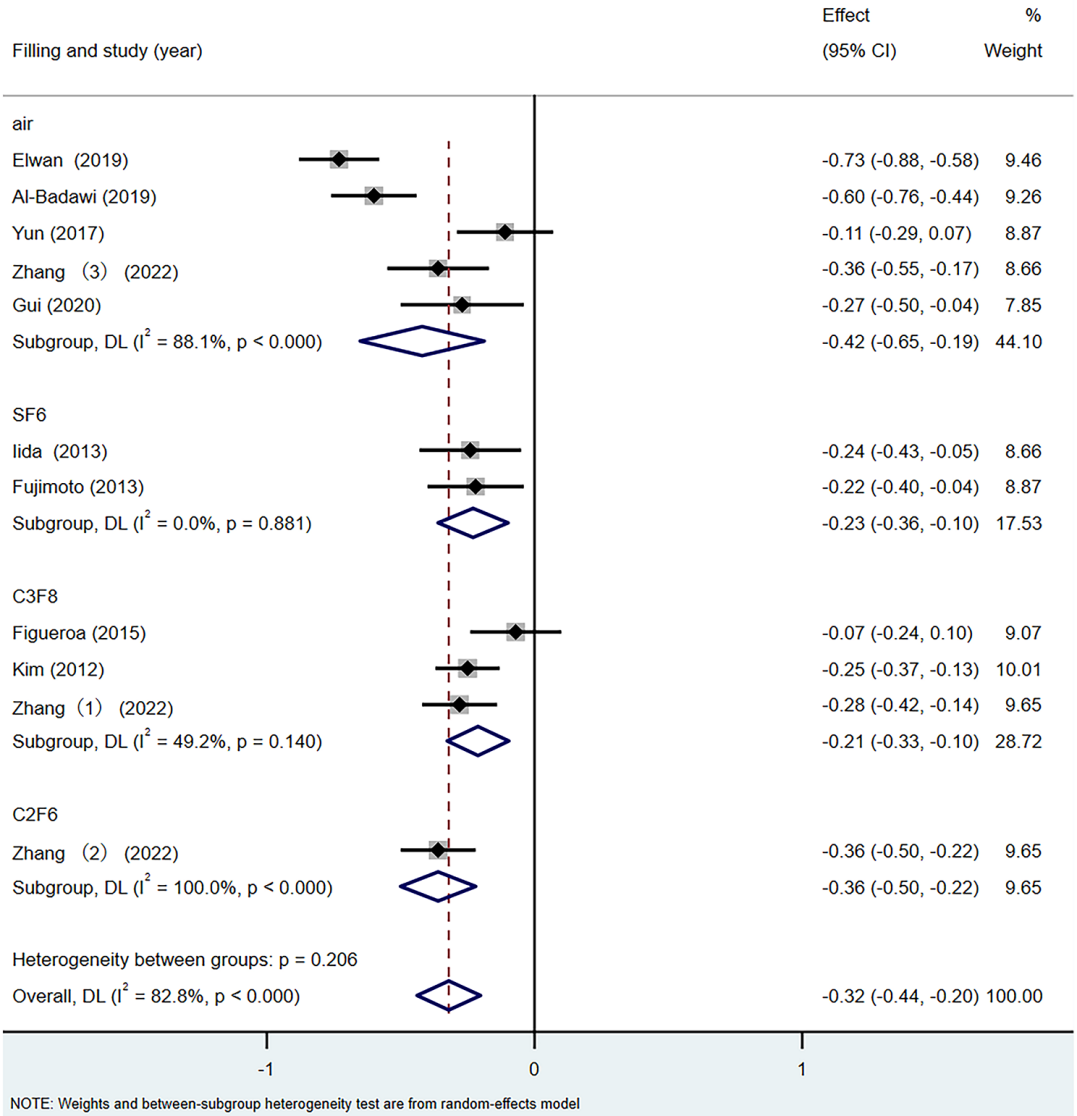
Forest map of BCVA Difference under different filling materials

According to the above analysis results, there was heterogeneity in the BCVA difference among different studies (I^2^ = 82.8%). Therefore, according to the different surgical filling materials, the BCVA difference was divided into the air group, the SF_6_ group, the C_3_F_8_ group and the C_2_F_6_ group for subgroup analysis. The results are shown in Fig. 6. In all four subgroups, the mean postoperative BCVA was higher than before surgery (SMD = − 0.32, 95% CI: −0.44, − 0.20, *p* < 0.001). The inter-group heterogeneity test was statistically significant (Q = 20.45, *p* < 0.001), the heterogeneity was higher in the air group (I^2^ = 88.1%), in the SF_6_ group (0%) and in the C_3_F_8_ group (49.2%). These results indicate that the difference in filling materials has an impact on the BCVA difference results and is one of the sources of heterogeneity.

**Fig. 7:**
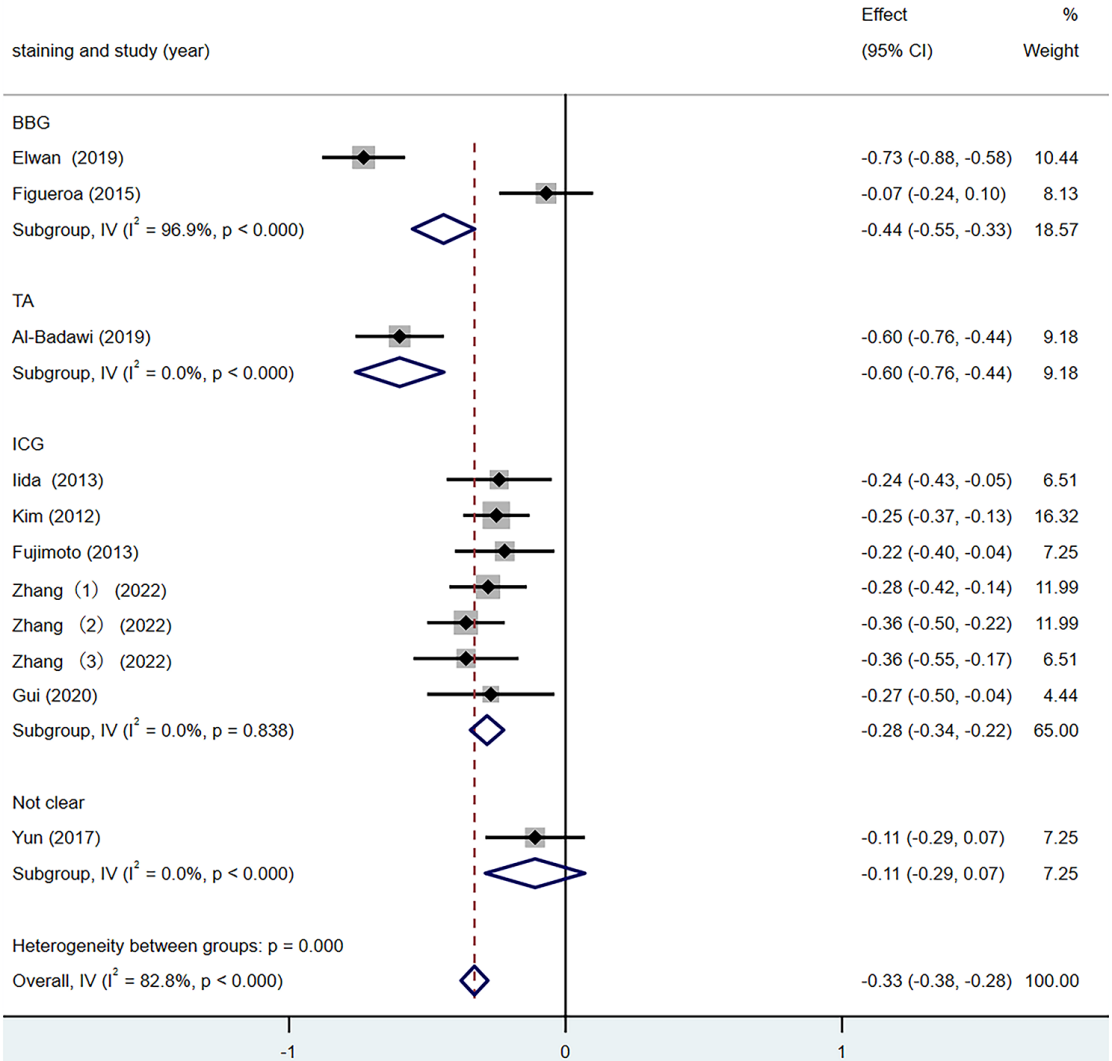
Forest map of BCVA Difference under different staining method

According to the different ILM dyeing materials, they were divided into the BBG group, the TA group and the ICG group for subgroup analysis. The results are shown in Fig. 7. In all three subgroups, the mean postoperative BCVA was higher than before surgery (SMD = − 0.33, 95% CI: −0.38, − 0.28, *p* < 0.001). The inter-group heterogeneity test was statistically significant (Q = 22.69, *p* < 0.001), the heterogeneity was higher in the BBG group (I^2^ = 96.9%) and 0% in the ICG group. These results indicate that ILM dyeing material is one of the sources of heterogeneity of the BCVA difference.

### Postoperative best-corrected visual acuity improvement rate

**Fig. 8:**
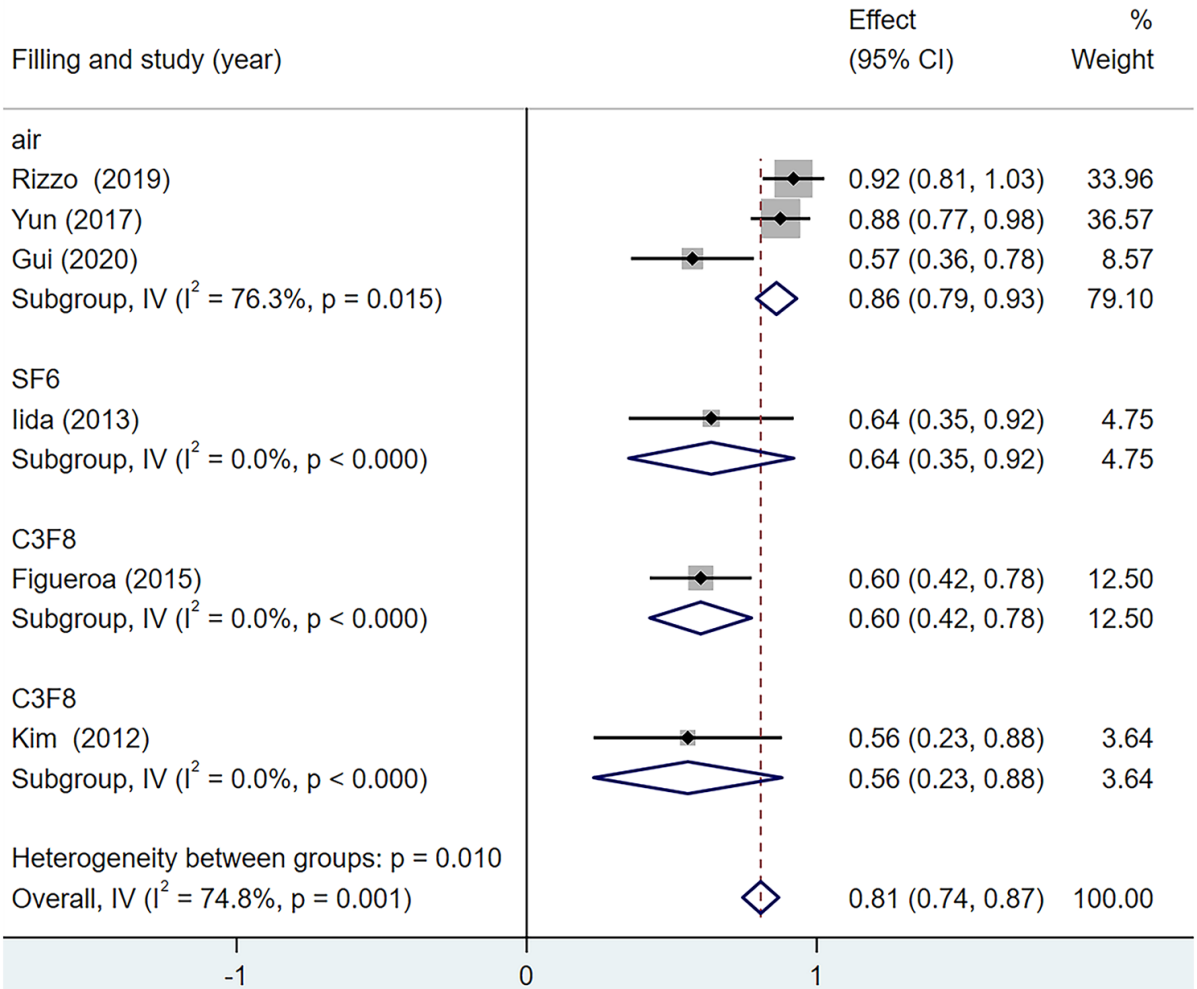
Forest map of Postoperative BCVA Improvement Rate under different filling materials

According to the above analysis results, there is heterogeneity in the BCVA improvement rate in various studies (I^2^ = 74.8%). Therefore, the postoperative BCVA improvement rate was categorised into the air group, the SF_6_ group and the C_3_F_8_ group for subgroup analysis according to the different surgical filling materials. As Zhang et al. [17] did not report rates for each subgroup, they were not included in the analysis. The results are shown in Fig. 8. Among the three subgroups, the inter-group heterogeneity test had statistical significance (Q = 11.33, *p* = 0.003), among which, the heterogeneity of the air group was higher (I^2^ = 76.3%), the SF_6_ group was 0% and the C_3_F_8_ group was 0%. This indicates that differences in filling materials influence the results of BCVA improvement rate in the postoperative period, which is one of the sources of heterogeneity.

**Fig. 9:**
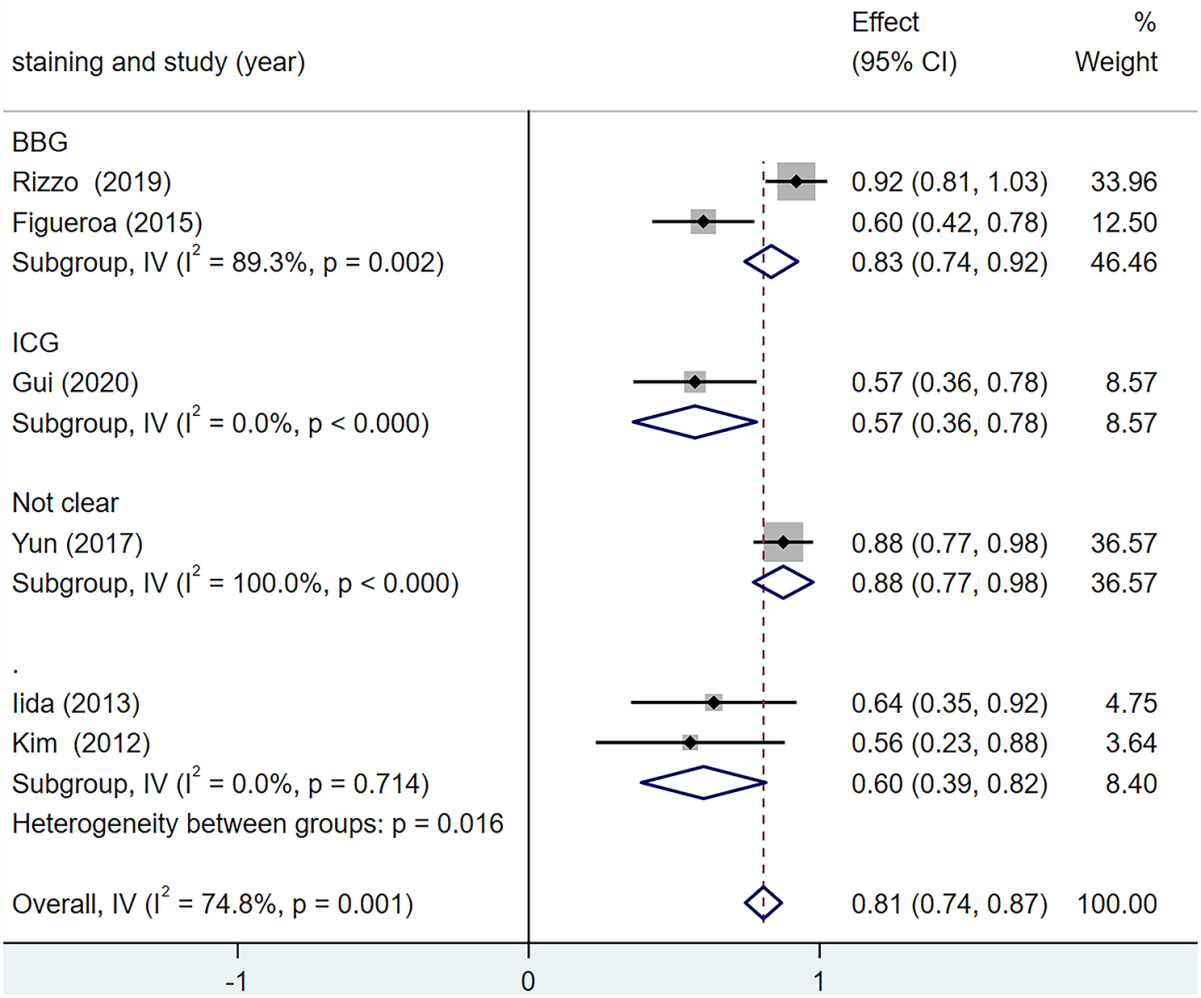
Forest map of Postoperative BCVA Improvement Rate under different staining method

According to the ILM dyeing materials, they were divided into the BBG group and the ICG group for subgroup analysis. The results are shown in Fig. 9. In the two subgroups, the inter-group heterogeneity test was statistically significant (Q = 10.30, *p* = 0.006), among which, the heterogeneity was higher in the BBG group (I^2^ = 89.3%) and 0% in the ICG group. These results indicate that ILM dyeing materials are one of the sources of postoperative BCVA improvement rate heterogeneity.

### Central foveal thickness difference

**Fig. 10:**
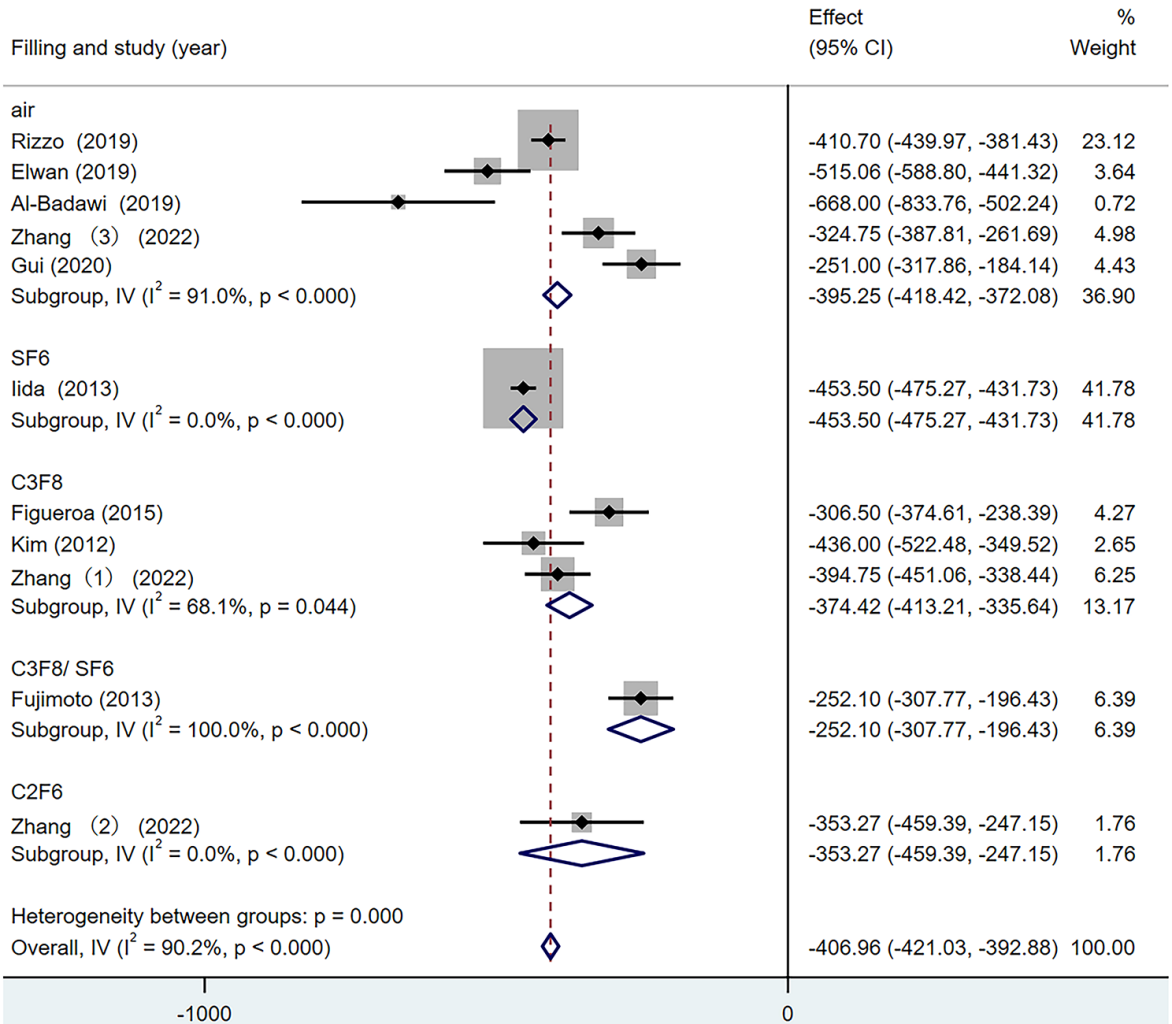
Forest map of CFT Difference under different filling materials

According to the above analysis results, there was heterogeneity in the CFT difference among studies (I^2^ = 90.2%). Therefore, according to the different surgical filling materials, groups were divided into the air group, the SF_6_ group, the C_3_F_8_ group and the C_2_F_6_ group for subgroup analysis. The results are shown in Fig. 10. Among the four subgroups, the inter-group heterogeneity test was statistically significant (Q = 8.34, *p* = 0.040), among which, the heterogeneity of the air group was 91.0%, the SF6 group was 0%, the C_3_F_8_ group was 68.1% and the C_2_F_6_ group was 0%. Subgroup analysis did not reduce the overall heterogeneity, so the effect of filling materials on the CFT difference was not reflected.

**Fig. 11:**
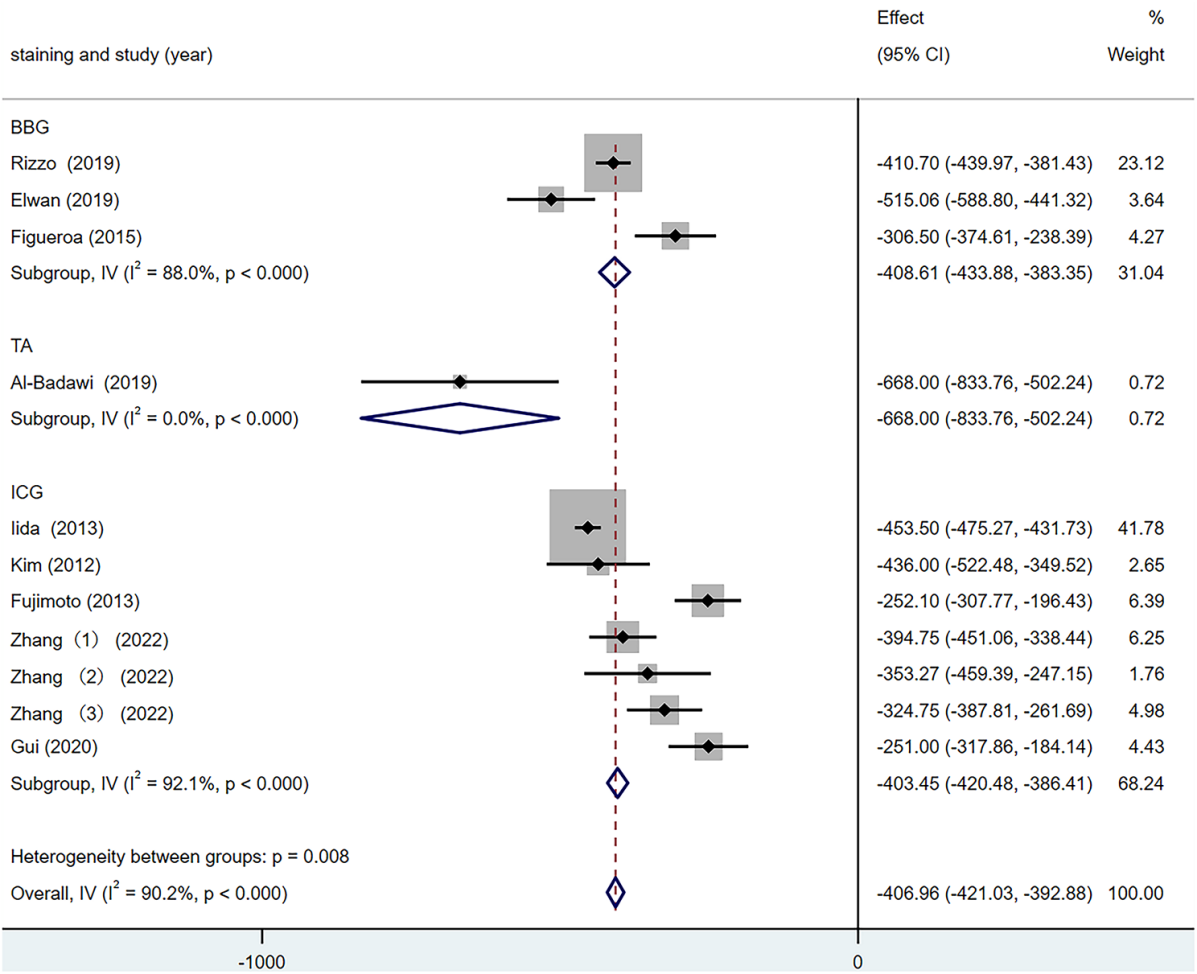
Forest map of CFT Difference under different staining method

According to the different ILM dyeing materials, they were divided into the BBG group, the ICG group and the TA group for subgroup analysis. The results are shown in Fig. 11. In the three subgroups, the inter-group heterogeneity test was statistically significant (Q = 9.71, *p* = 0.008), among which, the heterogeneity of the BBG group was 88.0%, and that of the ICG group was 92.1%. Subgroup analysis did not reduce the overall heterogeneity, so the effect of ILM staining materials on the CFT difference was not reflected.

### Publication bias

**Fig. 12:**
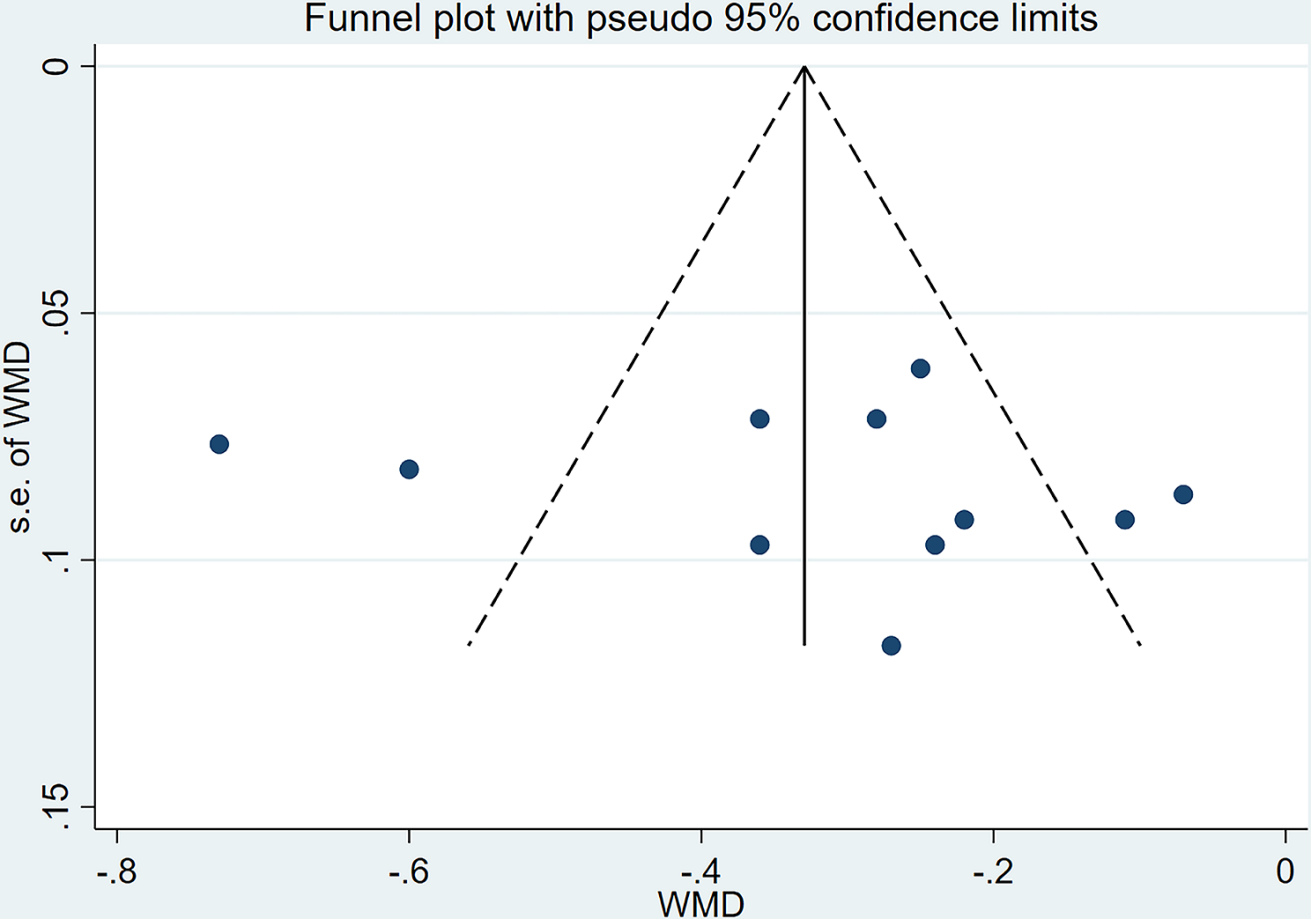
Funnel Plot of BCVA Difference

**Fig. 13:**
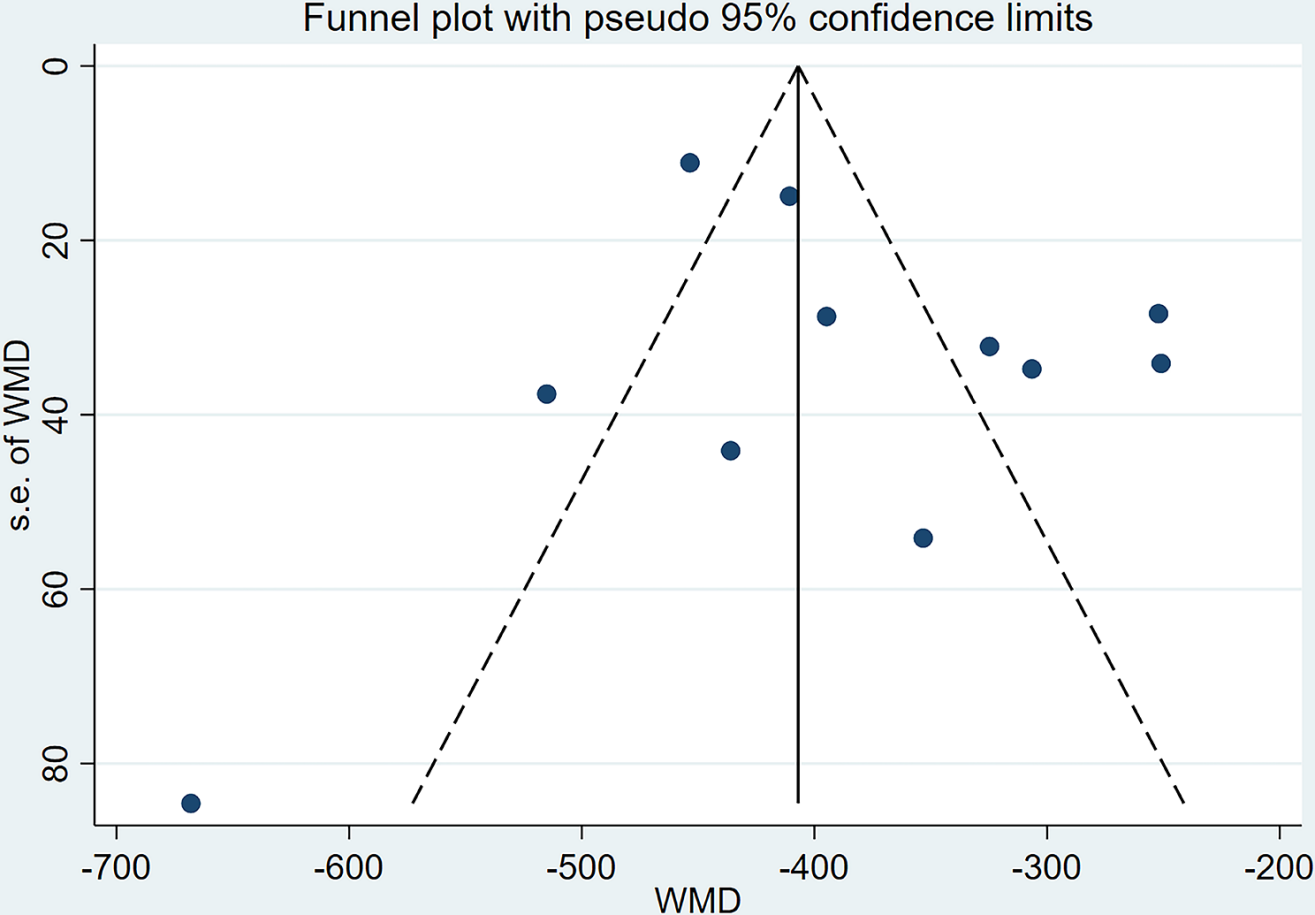
Funnel Plot of CFT Difference

An analysis of publication bias was conducted on studies reporting preoperative and postoperative BCVA and CFT differences. As shown in Figs. 12 and 13, studies reporting BCVA differences displayed some symmetry, combined with the Egger test result, (*t* = 0.49,*P* = 0.635 > 0.05), suggesting no significant bias. whereas those reporting CFT differences appeared more scattered, combined with the Egger test result, (*t* = 1.07,*P* = 0.312 > 0.05), suggesting no significant bias.

## Discussion

This meta-analysis aimed to evaluate the efficacy of PPV combined with ILM peeling and gas tamponade in the treatment of MF. The results demonstrated significant postoperative improvements in visual acuity and macular retinal structure.

Some studies suggest that increased cellular activity and proliferation at the vitreoretinal interface can lead to ILM hardening and reduced flexibility. Posterior vitreous detachment can create minute defects on the ILM, allowing glial cells to migrate to the retinal inner surface and subsequently differentiate into a premacular membrane. The residual vitreous cortex on the ILM can stimulate retinal growth factors, promoting tissue proliferation. Such proliferative changes anterior to the retina can exert tangential tractional forces on it. Combined with the reduced flexibility of the ILM, this can compromise the retina’s compliance [20–22]. Rey et al. collected ILM tissues from MF patients and found abundant collagen fibres and cell fragments on the inner surface of the ILM. This further confirms that cellular migration and collagen formation contribute to MF development. Additionally, the vitreous and ILM exert inward tractional forces on the retina, promoting the occurrence of macular foveoschisis [23]. Severe posterior staphyloma and sclerosed retinal vessels also accelerate the pathological process [24]. With the development of posterior staphyloma, pericentral vessels undergo sclerosis, limiting retinal stretching and promoting vascular separation from the ILM and other tissues [24]. Therefore, peeling the ILM can relieve traction on the retina, enhance retinal flexibility and accommodate an elongated eye axis and staphyloma.

The results of this study confirm that ILM peeling can effectively improve the visual acuity of MF patients and facilitate retinal repositioning. Retinal repositioning refers to the process of returning the retina from a detached or abnormal position to its normal anatomical position. Retinal detachment is the separation of the retinal neuroepithelial layer from the pigment epithelial layer and can be caused by a variety of reasons, including the following: retinal break – a full-thickness rupture of the retina, resulting in RD; retinoschisis – separation between the layers of the retina, but the entire retina is not ruptured; retinal traction – the retina is stretched and detached due to vitreous liquefaction or other reasons. These findings are consistent with several studies. For instance, Germano et al. reported a case of a patient with recurrent MF who initially underwent PPV combined with ERM peeling and was filled with C_3_F_8_. The patient remained stable for 17 months postoperatively but experienced a recurrence of MF with a significant decline in vision. However, after undergoing PPV combined with ILM peeling, there was no recurrence at the 6-month follow-up, and the BCVA improved to 20/25. An OCT examination revealed significant improvement in the macular structure [25]. Moreover, Sayanagi et al. described two cases of patients with refractory MF. One patient, after initial PPV treatment, did not show macular retinal repositioning even 9 months post-surgery. After undergoing PPV combined with ILM peeling, the foveal structure returned to normal within 1 month, and there was no recurrence of MF 24 months postoperatively. The BCVA improved from 20/100 preoperatively to 20/25. Another patient, despite undergoing two PPV procedures, continued to complain of decreasing vision. An OCT examination revealed persistent macular foveoschisis. After undergoing PPV combined with ILM peeling, foveal retinal repositioning was observed within 1 month. At the 22-month follow-up, the patient’s retinal structure and vision remained stable [26]. These studies attest that PPV combined with ILM peeling can more effectively and thoroughly eliminate the traction forces on the retina, providing an effective treatment for patients with recurrent and refractory MF.

Common postoperative complications of MF include cataracts, FTMH and MHRD. In this study, the incidence of cataracts reached 55.9%, which is related to changes in the intraocular environment and aqueous circulation after PPV, thereby accelerating and exacerbating cataract formation. This suggests that cataract extraction and intraocular lens implantation can be performed concurrently with PPV, particularly in elderly patients. Therefore, when considering surgical treatment, the patient’s cataract risk needs to be carefully assessed and, where appropriate, consideration should be given to concurrent cataract extraction and intraocular lens implantation with surgery. In addition, some researchers have discussed some potential risks during surgery, such as the risk of postoperative macular perforation, that may be caused by artificial endothelial peeling. Therefore, the potential complications of surgery need to be carefully considered and weighed against the benefits of surgery to provide the patient with the best treatment option. Some scholars debate the treatment of MF with PPV combined with LM peeling, mainly considering that artificial ILM peeling may lead to iatrogenic MH. Ho believes that the Muller cell cone (MCC) is an essential structure in the macular region and is crucial for binding photoreceptor cells. Damage to its structure is the mechanism for FTMH. Thus, if the MCC is injured during ILM peeling, subsequent MH may occur [27]. As a result, some researchers propose preserving the foveal inferior inner macular (IIM) during ILM peeling to maintain the integrity of the MCC in the macular region. A retrospective study by Sisk et al. suggests that the incidence of MH caused by foveal ILM peeling is much lower than that of complete macular ILM peeling (28.6% vs. 0%), achieving better postoperative visual acuity and macular retinal structure recovery [28]. Other studies indicate there is no significant difference between the two procedures in terms of postoperative visual improvement and CFT reduction [11]. Therefore, to reduce the risk of postoperative complications, a new, safe method of preserving foveal ILM during IIM peeling can be adopted. However, its operation is relatively challenging, and whether its therapeutic effect is better than complete peeling remains to be further explored.

Although this study has provided valuable insights, several limitations warrant consideration. First, the reliance on predominantly retrospective studies introduces inherent variability in surgical methodologies, potentially impacting result consistency. Future randomised controlled trials are essential for standardising procedures and prospectively evaluating surgical efficacy and safety. Second, the subjective determination of vitreous cavity filling material by surgeons may introduce bias into the results. Third, the inconsistency in final follow-up times leads to disparate outcome measurements, highlighting the importance of uniform time points for comparison. Fourth, the stage or severity of foveoschisis, the classification system and the proportion of foveoschisis of different severity will have different effects on surgical outcomes. This may affect preoperative and postoperative visual acuity, thereby leading to differences in surgical outcomes, which reduces the extrapolability of the research results. Future research with stricter publication standards and comprehensive reporting is needed to validate these findings.

Based on the results of this study, it is evident that in the early stages of MF, before complications arise, the use of PPV combined with LM vitrectomy with gas tamponade can effectively treat MF. After the procedure, patients’ BCVA and CFT show significant improvement, with an average of 90% of patients achieving the disappearance of the cleavage cavity and complete flattening of the macular retinal area. Our study found that PPV combined with ILM vitrectomy and gas tamponade for the treatment of MF, MH and RD had a lower mean incidence of serious postoperative complications. Although this is a promising trend, comparison with studies reporting higher complication rates with other treatments is needed to fully assess the comparative advantage of this approach; the generalisability of the results requires attention to allow more comprehensive conclusions to be drawn. For example, changes in patient demographic characteristics across studies, such as age, disease severity and medical history, which may affect the generalisability of surgical outcomes, should be examined. An analysis should be carried out of potential differences in different study settings, such as different healthcare settings, where there may be different clinical practices and operating procedures. Finally, other surgical methods may produce different results and need to be fully explored.

As anticipated, the incidence of cataracts was relatively high in this study population, although the specific factors influencing cataract development in our study population warrant further investigation. This finding underscores the need for further in-depth clinical research to identify the specific risk factors and potential preventive strategies to manage cataract development after vitrectomy.

## Data Availability

All data generated or analyzed during this study are included in this published article.
